# Isolated Brain Trauma in Cats Triggers Rapid Onset of Hypovolemia

**DOI:** 10.1007/s12028-016-0348-5

**Published:** 2016-11-07

**Authors:** Peter Bentzer, Per-Olof Grände

**Affiliations:** 1 Department of Clinical Sciences, Anesthesiology and Intensive Care, Helsingborg Hospital, Lund University, Lund, Sweden; 2 Department of Clinical Sciences, Anesthesiology and Intensive Care, Lund University Hospital, Lund University, 22185 Lund, Sweden

**Keywords:** Traumatic brain injury, Plasma volume, Fluid percussion trauma, Intracranial pressure, Blood pressure, Vascular leak

## Abstract

**Background:**

Hemodynamic instability responsive to fluid resuscitation is common after a traumatic brain injury (TBI), also in the absence of systemic hemorrhage. The present study tests if an isolated severe TBI induces a decrease in plasma volume (PV).

**Methods:**

The study was performed in three groups of anesthetized and tracheostomized male cats (*n* = 21). In one group (*n* = 8), the cats were prepared with a cranial borehole (10 mm i.d) used to expose the brain to a fluid percussion brain injury (FPI) (1.90–2.20 bar), and two smaller cranial boreholes (4 mm i.d) for insertion of an intracranial pressure (ICP) and a microdialysis catheter. To differentiate the effect of FPI from that of the surgical preparation, a sham group was exposed to the same surgical preparation but no FPI trauma (*n* = 8). A control group had no brain trauma and no surgical preparation (*n* = 5). PV was determined by a ^125^I-albumin dilution technique. PV, electrolytes, pH, BE (base excess), hematocrit (Hct), P_a_O_2_, and P_a_CO_2_ were measured at baseline and after 3 h. Mean arterial pressure (MAP) was measured continuously. ICP was measured in the FPI and the sham group.

**Results:**

In the FPI group, PV decreased by 11.2 mL/kg from 31.7 mL/kg (*p* < 0.01) with a simultaneous increase in Hct and decrease in pH. In the sham group, PV decreased by 5.7 mL/kg from 32.7 mL/kg (*p* < 0.01). The control group showed no PV reduction.

**Conclusions:**

The results support that an isolated severe head trauma triggers a significant and rapid reduction in PV, most likely due to vascular leak.

## Introduction

Brain damage following a TBI is both an initial direct mechanical injury and a subsequent secondary brain injury [1, 2]. The secondary injury includes an inflammatory response with release of a host of cytokines and a profound acute-phase response, which is thought to contribute to disruption of the blood–brain barrier and brain edema [2–7]. Release of inflammatory substances from the brain may also cause a systemic inflammatory response syndrome (SIRS) with a general increase in transcapillary leak of fluid and proteins and development of hypovolemia [8–10]. A low PV may lead to adverse effects due to reduced cardiac output and activation of the baroreceptor reflex with increased sympathetic discharge and release of catecholamines, resulting in general vasoconstriction also including the brain [11]. Especially, injured parts of the brain suffering compromised circulation may be uniquely sensitive to hypovolemia [11].

The hypothesis that an isolated TBI may induce systemic inflammation with systemic transcapillary leak of albumin is supported by the observation that albumin concentration in plasma is commonly decreased shortly after a TBI [12]. The clinical experience that fluid administration often restores hemodynamic stability in patients with TBI, also in the absence of major *systemic* hemorrhage, is compatible with hypovolemia [13, 14]. Plasma volume substitution is also an important component in modern head trauma guidelines [15, 16].

In spite of the fact that significant hypovolemia may develop in TBI and clinical signs are compatible with hypovolemia, there are no data available on the development and significance of hypovolemia in the setting of isolated TBI.

The present study on the anaesthetized cat tested the hypothesis that an isolated TBI in terms of a standardized fluid percussion brain injury (FPI) [17–19] without intracranial hemorrhage is associated with a reduction in PV. To separate effects of the brain trauma from those induced by the surgical preparation, three groups of animals were studied; one group subjected to the surgical preparation followed by a brain trauma, one group subjected only to the surgical preparation (no brain trauma), and one control group exposed only to tracheostomy and arterial and venous cannulation.

## Materials and Methods

### Anesthesia and Experimental Procedure

The study was approved by the Ethics Committee for Animal Research at Lund University, Sweden (application no M61-12). The animals were treated in accordance with the* Guidelines for Care and Use of Laboratory Animals* of the National Academy of Sciences. Twenty-one adult male cats weighing 3.7–5.0 kg (4.4 ± 0.4 kg) were included in the study. The cats were given food up to 12 h before and water up to 1 h before the start of the experiment. There was no significant difference in weight between the three groups. Anesthesia was induced with ketamine (4 mg/kg) given intramuscularly. A catheter was inserted into a subcutaneous vein of the left forelimb, and anesthesia was continued using alpha-chloralose (50 mg/kg bolus followed by an infusion of 4 mg/kg/h) given intravenously. After tracheostomy, the animals were ventilated with air using a volume-controlled ventilator (Ugo Basile, Comerio-Varese, Italy), maintaining an approximately constant end-tidal PCO_2_ within the normal range of 36–40 mmHg (4.8–5.3 kPa) (CO_2_ Analyzer; MedAir, Delsbo, Sweden). The animals were placed on a heating pad, and body core temperature was kept constant at 37.3–37.6 °C through a feedback circuit controlled by continuous measurement via a temperature probe inserted in the esophagus. The animals were given 5 mL/kg/h of 0.9% NaCl intravenously as fluid substitution starting after initiation of anesthesia and lasting up to the end of the experiment. Mean arterial blood pressure (MAP) was recorded continuously throughout the experiment via a catheter inserted into the left brachial artery and filled with heparinized fluid. Arterial blood samples were taken from this catheter.

### Fluid Percussion Brain Injury (FPI)

**Fig. 1:**
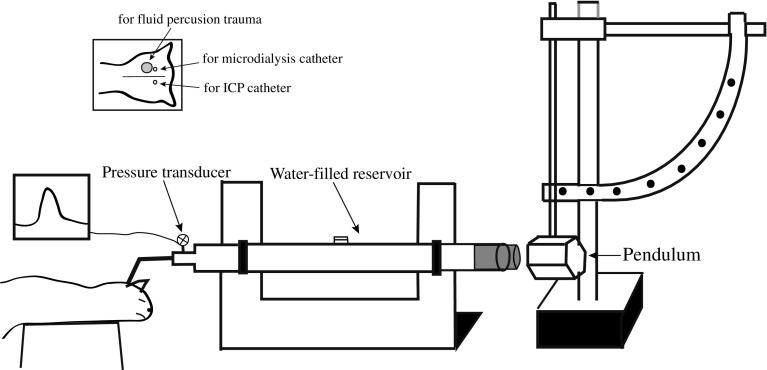
Schematic illustration of the fluid percussion model of mechanical brain injury. A metal adaptor with a hole of 8 mm in diameter was fixed in the cranium and was in fluid connection via a metal tubing to a Plexiglass cylinder filled with physiological saline free from air bubbles. The other end of the cylinder is occluded by a Plexiglass cork mounted on O-rings. Injury is produced by striking the cork with a 4.4-kg pendulum dropped from a known fall height. The pressure in the cylinder is recorded via an extracranial pressure transducer on a screen

A traumatic brain injury was induced using a FPI model as described previously [17–19]. Briefly, after a midline scalp incision over the parietal bone, the scalp and the temporal muscles were dissected free from the cranium in a circular area of about 2.5 cm in diameter. A borehole of 10 mm in diameter used for the FPI trauma was drilled in the skull just to the left of the midline down to the dura. The dura was intact in all the animals. One borehole with a diameter of 4 mm was drilled close to the 10-mm hole down to the dura, and another 4-mm borehole was drilled to the right of the midline (Fig. 1). The scalp incision and drilling of the boreholes only resulted in minor bleeding. A 10-mm metal adapter with an external screw thread and an inner hole of 8 mm in diameter was screwed into the large hole and further attached to the skull bone with dental cement. The animals were now connected to the trauma device via the metal adaptor, after assuring that the whole fluid system from the piston to the dura was free from air (Fig. 1). The piston was hit by a 4.4-kg pendulum from a specific fall height, producing a brief pressure pulse (25 ms) via the fluid column onto the exposed dura and underlying brain. A pressure transducer connected to the fluid column measured the applied pressure (Fig. 1). Peak pressures were found to be in the range of 1.90–2.20 bars (2.05 ± 0.13 bar).

After injury, the metal adaptor was removed from the scalp. A microdialysis catheter with an external diameter of 0.5 mm and length of 10 mm was placed via the small left borehole (CMA/20; CMA Microdialysis, Stockholm, Sweden) (Fig. 1) in the cortex. An ICP-measuring parenchymal transducer (Codman and Shurleff, Raynham, MA) was inserted in the brain 6–8 mm below the dura via the small right borehole (Fig. 1). All the boreholes were then sealed with histacryl to prevent leakage of cerebrospinal fluid and bleeding from the bone borders. Due to technical failure in the analysis of the microdialysis samples, no microdialysis data can be presented.

### Plasma Volume (PV)

PV was determined with a well-established technique [20–23] by measuring the increase in radioactivity in 500 µL of plasma taken 5 min after an intravenous injection of human ^125^I-albumin with a known amount of activity. The increase in radioactivity was calculated by subtracting the activity in a blood sample taken just before the injection from that taken 5 min after the injection. Each PV measurement was thereby adjusted for remaining radioactivity from previous measurements. To calculate the amount of radioactivity given, the remaining radioactivity in the emptied vial, syringe, and needle used was measured and subtracted from the total radioactivity in the prepared dose. This is a reliable and established technique for PV measurement with small sources of error and reproducible results [20, 21, 23] (see “Discussion” section). Free iodine following precipitation with 10% trichloroacetic acid was measured regularly and was found to be less than 1.6% in all experiments. Radioactivity was measured with a gamma counter (Wizard 1480; LKB-Wallace, Turku, Finland). Plasma volume in blood samples taken to measure PV and electrolytes from the start to the end of the experiment was calculated to be about 0.6 mL/kg.

### Protocol

The study was performed in three randomized groups of animals. One group called “the FBI group” was exposed to a FPI trauma as described above. Another group called “the sham group” was exposed to the same experimental procedures as in “the FBI group” (anesthesia, cranial boreholes, arterial and venous cannulations, and tracheostomy) but was not exposed to a brain trauma. A third group called “the control group” was anesthetized, cannulated, and tracheostomized as in the other groups; but there was no trauma in terms of scalp incision, cranial boreholes, and FPI.

PV was measured at baseline in the three groups. It was also measured 3 h after trauma in “the FBI group” and at corresponding time point for “the sham group” and “the control group.” Sodium/s, potassium/s, P_a_O_2_, P_a_CO_2_, pH, base excess (BE), and hematocrit (Hct) were measured from arterial blood samples taken at baseline and at the end of the experiment 3 h later. MAP was measured continuously from the start to the end of the experiment in all groups. ICP was measured in “the FBI group” and “the sham group” from the start to the end of the experiment. MAP and ICP were recorded on a Grass polygraph. After finishing the experiment, the cats were euthenized with an intravenous bolus infusion of 3 M KCl.

### Statistical Analysis

Data are presented as mean ± SD. Student’s *t* test for paired observations was used for comparison of PV, Hct, pH, P_a_O_2_, P_a_CO_2_, BE, and electrolytes at baseline and at the end of the experiments for all three groups. Student’s *t* test for unpaired observations was used for comparing PV reduction in the FBI and the sham groups. Alterations in MAP and ICP were analyzed with a nonparametric Friedman test followed by post hoc testing using a Wilcoxon–Neyman–McDonald–Thompson test. All data except data for MAP and ICP were normally distributed. Statistical calculations were made using the computer software R (version 3.0.0) for Linux.

## Results

### Physiological Parameters

Data for arterial pH, Na^+^/s, K^+^/s, BE, and Hct for the three groups are presented in Table 1. Arterial pH decreased in the FBI group (*p* < 0.05), but there was no change in pH in “the sham” and “the control group.” Na^+^/s, K^+^/s, P_a_O_2_, and P_a_CO_2_ did not change in any group. Hct increased from baseline to 3 h after trauma in “the FBI group” (*p* < 0.05), while there was just a tendency to increase in “the sham group” (*p* = 0.08). Hct was unchanged in “the control group.”

**Table 1:** Data (mean ± SD) for pH, P_a_O_2_, P_a_CO_2_, sodium, potassium, base excess, and hematocrit (Hct) for “the fluid percussion (FBI) group,” “the sham group,” and “the control group”

	pH	PaO2 (kPa)	PaCO2 (kPa)	Sodium (mmol/L)	Potassium (mmol/L)	Base excess	Hct (%)
*FBI group*							
Baseline	7.32 ± 0.04	11.5 ± 0.7	4.9 ± 0.3	152 ± 1	3.6 ± 0.3	−6.0 ± 1.8	35 ± 3
End of exp.	7.25 ± 0.04*	10.9 ± 1.3	5.1 ± 0.1	147 ± 3	4.3 ± 1.0	−10.1 ± 1.7*	51 ± 5
*Sham group*							
Baseline	7.30 ± 0.05	11.1 ± 0.8	4.5 ± 0.7	153 ± 2	3.4 ± 0.3	−7.5 ± 1.9	34 ± 2
End of exp.	7.32 ± 0.06	11.4 ± 0.7	4.5 ± 0.4	151 ± 2	3.8 ± 0.6	−9.0 ± 1.9	40 ± 5
*Control group*							
Baseline	7.34 ± 0.06	10.6 ± 1.3	4.4 ± 0.4	152 ± 2	3.4 ± 0.2	−6.6 ± 1.5	37 ± 4
End of exp.	7.34 ± 0.05	11.0 ± 1.2	4.4 ± 0.3	152 ± 3	3.3 ± 0.2	−6.4 ± 1.5	36 ± 5

* *p* < 0.05 compared to baseline

Data for MAP and ICP are presented in Table 2. There was an increase in MAP from 122 ± 9 at baseline to 136 ± 13 mmHg at 3 h after trauma in “the FBI group” (*p* < 0.05), whereas it tended to decrease in the “sham group” (*p* = 0.07). MAP did not change in “the control group.” ICP increased from 10.4 ± 3 mmHg at baseline to 15.0 ± 4 mmHg at 3 h after trauma in “the FBI group” (*p* < 0.05). There was no change in ICP in “the sham group.”

**Table 2:** Data (mean ± SD) for mean arterial blood pressure (MAP, in mmHg) at baseline and 1, 2, and 3 h after baseline for “the fluid percussion” (FBI) group,” “the sham group,” and “the control group.” Data (mean ± SD) for intracranial pressure (ICP, in mmHg) at baseline and 1, 2, and 3 h after baseline are presented for “the FBI group” and “the sham group”

	Baseline	1 h after baseline	2 h after baseline	3 h after baseline
*FBI group*				
MAP (in mmHg)	122 ± 9	134 ± 15	134 ± 20	135 ± 13*
ICP (in mmHg)	10.4 ± 3	13.0 ± 6	14.9 ± 5	15.0 ± 4*
*Sham group*				
MAP (in mmHg)	119 ± 19	108 ± 21	115 ± 24	112 ± 21
ICP (in mmHg)	9.6 ± 1.5	9.6 ± 2.1	10.5 ± 2.6	10.4 ± 1.8
*Control group*				
MAP (in mmHg)	120 ± 17	122 ± 22	122 ± 24	124 ± 23

**Fig. 2:**
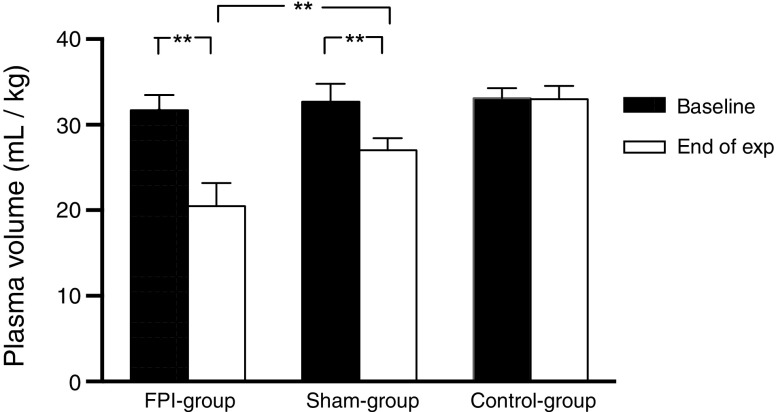
Plasma volume (PV) at baseline and at the end of the experiment 3 h later for the 3 groups (“the “fluid percussion (FBI) group” + “the sham group,” and “the control group”). Student’s *t* test for paired observations was used for comparison of PV at baseline and at the end of the experiments for each group. Student’s *t* test for unpaired observations was used for comparing PV reduction between “the FBI group” and “the sham group” (**p < 0.01)

Data for PV for the three groups are presented in Fig. 2. PV decreased from 31.7 ± 1.7 mL/kg at baseline to 20.5 ± 2.7 mL/kg at 3 h after the trauma in “the FBI group” (*p* < 0.01). In “the sham group” (exposed only to surgical preparation), PV decreased from 32.7 ± 2.0 mL/kg at baseline to 27.0 ± 1.4 mL/kg at the end of the experiment (p < 0.01). PV at the end of the experiment differed significantly between “the FBI group” and “the sham group” (*p* < 0.01). In “the control group,” plasma volume was 33.1 ± 1.2 mL/kg at baseline and 33.0 ± 1.5 mL/kg at the end of the experiment (ns).

## Discussion

The present study on the cat showed that an isolated brain trauma resulted in a significant decrease in PV, with a simultaneous increase in Hct, and this in spite of a continuous infusion of 5 mL/kg/h of saline. It also showed that only about half of the PV reduction could be attributed to the FBI brain trauma, while the rest of the trauma was associated with the preparation. ICP and MAP increased after the FBI trauma, whereas no significant change in MAP could be detected in “the sham group” or in “the control group.” There was no change in ICP in “the sham group.” There were no alterations in P_a_O_2_, P_a_CO_2_, Na^+^/s, or K^+^/s in any group. pH and BE decreased in “the FBI group.” Unchanged P_a_CO_2_, simultaneously with a decrease in BE, means that the lowered pH in the FPI group is of metabolic nature.

The ^125^I-dilution technique for measurement of PV is a well-established and reliable method in experimental and clinical studies for both normal and inflammatory states [20–24]. There may have been some overestimation of measured PV because of transcapillary escape of radioactive albumin during the 5-min period between injection of the tracer and collection of the blood sample. Further, free iodine in the injected tracer is distributed quickly to the entire extracellular space, which can result in some overestimation; but the proportion of free iodine was small in this study (<1.6%). We conclude that possible sources of error are small and similar in all groups and therefore will not influence the conclusions made below.

The peak FPI trauma was in the range of 1.9–2.2 bars. A previous study on cat using a similar fluid percussion device [17] showed that a peak trauma below about 2.2 bars caused only minimal microscopic subarachnoidal and contusional hemorrhage and EEG changes similar to those observed in human trauma [17, 25].

Due to the rigid cranium, there is no space for intracranial expansion. Total brain weight of the cat is about 40 g, and total cerebrospinal volume and intracranial blood volume are less than 2 mL/kg. This means that just a very small part of the observed loss in PV can be explained by an intracranial bleeding and swelling of the brain, also supported by the relatively small increase in ICP (Table 2). The simultaneous increase in Hct and decrease in PV, therefore, most likely can be explained by a systemic loss of plasma.

As mentioned, traumatic brain injury triggers an inflammatory response, which is thought to contribute to the secondary injuries and to brain edema [2–7, 17]. We hypothesized that a brain trauma induced a systemic inflammation, which may induce a systemic vascular leak and contribute to the observed decrease in PV [12, 26]. Previous experimental studies have shown that brain trauma initiates a cascade of multiple anti- and proinflammatory pathways with permeability-increasing properties, such as release of various cytokines such as IL6, TNF alpha, and IL10 [8, 26]. Some further proinflammatory substances and mechanisms associated with a head injury are the release of chemokines, prostaglandins, and free radicals [4, 5, 27–29]. Also the existence of a general hyperadrenergic state with increase in sympathetic discharge may contribute to PV leak via effect on blood pressure or indirectly via endothelial effects [28, 29]. So far, however, no specific anti-inflammatory drug or blockade of inflammatory pathways has shown beneficial effects on outcome in clinical studies in head injury [1, 27, 30, 31].

Our results of an increase in MAP following brain trauma despite a significant reduction in PV most likely are secondary to a trauma-induced increase in sympathetic activity and catecholamine release [28, 31, 32]. An increase in blood pressure after a head trauma was also found in the previous fluid percussion study on the cat [17]. Increases in blood pressure are also common after a head trauma in man [32].

Both clinical and experimental studies have demonstrated that an increase in arterial blood pressure increases vascular leak of fluid and macromolecules, particularly in inflammatory states and most likely due to an increase in microvascular hydrostatic pressure [33, 34]. Based on this, it could be speculated that part of the decrease in PV seen in “the FBI group” may be an effect of increase in blood pressure.

To evaluate the loss in PV caused by the brain trauma, the amount of PV loss in “the sham group” must be subtracted from the PV loss in “the FBI group.” We found that about half of the total loss in PV in the present study was a consequence of the surgical preparation and half of the brain trauma.

Our finding that brain trauma induces a decrease in PV means that hypovolemia is a potential contributor of reduced cerebral circulation and secondary brain injuries, especially in areas with compromised blood flow from start, such as the penumbra zone [11]. If applicable to man, hypovolemia should be considered in patients after a traumatic brain injury. That the hypovolemia is not reflected in a decrease in blood pressure agrees with previous studies and suggests that blood pressure alone is a poor resuscitation endpoint in brain trauma [35, 36].

The brain trauma in man is different from a fluid percussion brain trauma. It was standardized in the present study and does not reflect the case mix and variability of head trauma in clinical practice, and it includes cranial burr holes. The inflammatory response after a head injury may also be different between cat and man. Loss of plasma in man after a head trauma finds support by the fact that albumin concentration in plasma is lowered, most likely due to transcapillary leak [12, 15], and that plasma fluid substitution is of importance to preserve hemodynamic stability [2, 13]. Cranial boreholes in man are much smaller relative to the size of the cranium than those in the present study on the cat and, hence, the borehole trauma is proportionally smaller in man. If borehole trauma influences PV loss, as indicated from the present results, the effect of burr holes on PV is likely to be less pronounced in man than in the cat. However, it is possible that larger cranial openings in patients, such as those performed for decompressive craniotomy or for evacuation of a large subdural hematoma, may influence PV.

In conclusion, to our knowledge, this is the first study that specifically shows an isolated severe head trauma resulting in a relatively rapid loss of PV, most likely due to a general transcapillary leak, with preserved or even increased MAP. If applicable to man, the results suggest that brain trauma may result in hypovolemia unrecognized by a lowered MAP, which could contribute to poor outcome.
